# Attempts to Target *Staphylococcus aureus* Induced Osteomyelitis Bone Lesions in a Juvenile Pig Model by Using Radiotracers

**DOI:** 10.3390/molecules25184329

**Published:** 2020-09-21

**Authors:** Pia Afzelius, Aage Kristian Olsen Alstrup, Ole Lerberg Nielsen, Karin Michaelsen Nielsen, Svend Borup Jensen

**Affiliations:** 1 Department of Nuclear Medicine, Aalborg University Hospital, 9100 Aalborg, Denmark; karinmn007@hotmail.com (K.M.N.); svbj@rn.dk (S.B.J.); 2 North Zealand Hospital, Copenhagen University Hospital, 3400 Hillerød, Denmark; 3 Department of Nuclear Medicine and PET, Aarhus University Hospital, 8200 Aarhus, Denmark; aagealst@rm.dk; 4 Department of Clinical Medicine, Aarhus University, 8200 Aarhus, Denmark; 5 Department of Veterinary and Animal Sciences, Faculty of Health and Medical Sciences, University of Copenhagen, 2000 Copenhagen F, Denmark; olelerbergnielsen@gmail.com; 6 Department of Chemistry and Biochemistry, Aalborg University, 9100 Aalborg, Denmark

**Keywords:** osteomyelitis, animal experimentation, pigs, PET/CT, [^18^F]FDG, [^18^F]NaF, [^68^Ga]Ga-DOTA-K-A9, [^68^Ga]Ga-DOTA-GSGK-A11 [^68^Ga]Ga-DOTA-Siglec-9, [^68^Ga]Ga-ubiquicidin

## Abstract

Background [^18^F]FDG Positron Emission Tomography cannot differentiate between sterile inflammation and infection. Therefore, we, aimed to develop more specific radiotracers fitted for differentiation between sterile and septic infection to improve the diagnostic accuracy. Consequently, the clinicians can refine the treatment of, for example, prosthesis-related infection. Methods: We examined different target points; *Staphylococcus aureus* biofilm (^68^Ga-labeled DOTA-K-A9 and DOTA-GSGK-A11), bone remodeling ([^18^F]NaF), bacterial cell membranes ([^68^Ga]Ga-Ubiquicidin), and leukocyte trafficking ([^68^Ga]Ga-DOTA-Siglec-9). We compared them to the well-known glucose metabolism marker [^18^F]FDG, in a well-established juvenile *S. aureus* induced osteomyelitis (OM) pig model. Results: [^18^F]FDG accumulated in the OM lesions seven days after bacterial inoculation, but disappointingly we were not able to identify any tracer accumulation in OM with any of the supposedly more specific tracers. Conclusion: These negative results are, however, relevant to report as they may save other research groups from conducting the same animal experiments and provide a platform for developing and evaluating other new potential tracers or protocol instead.

## 1. Introduction

*Staphylococcus aureus* (*S. aureus*) causes skin, soft tissue, bone, pleuropulmonary infections, infective endocarditis, sepsis, osteoarticular, and device-related infections [1]. To prevent disease progression and to reduce the number of complications, it is essential to diagnose bacterial infections at an early stage and initiate the best therapy. As stated by the WHO and others, the annual consumption of antibiotics is increasing, and the support for its use is often insignificant [2,3]. The prevalence of specific Multi-Drug Resistant (MDR) bacteria is associated with the usage of broad-spectrum antibiotics, both for empiric as well as for definite therapy [4]. According to a recent report, more than 2.8 million antibiotic-resistant infections occur in the U.S. each year, and more than 35,000 people die as a result [5]. To use the correct treatment, clinicians need to identify the location of inflammation and discriminate between sterile lesions and infections by pathogenic microorganisms. To fit this purpose, nuclear medicine imaging techniques in combination with Computed Tomography (CT) provide valuable information on functional and anatomical data. These imaging techniques visualize the extent, the activity, the size, and the location of the disease. The molecular imaging techniques of nuclear medicine, such as scintigraphy and Positron Emission Tomography (PET), are characterized by visualizing the whole body.

However, the commonly used tracer [^18^F]-fluorodeoxyglucose ([^18^F]FDG) and [^111^In]-labeled leukocytes cannot distinguish between sterile inflammation and infection. Osteomyelitis is a severe bone infection, and the overall purpose of our project was to identify radiotracers that may improve the scanning of the bacterial infection. We have, therefore, searched for other tracers; tracers made for targeting the *S. aureus* biofilms or the infected bone lesion itself.

^68^Ga-labeled PET tracers are of particular interest. This radiopharmaceutical is nontoxic. The half-life of 68 minutes makes it suitable for peptide pharmacology. ^68^Ga is available from ^68^Ge/^68^Ga generators and independent of reliance on local cyclotron production [6]. In vitro specificity for bacterial binding and in vivo stability with favorable kinetics makes these radiotracers candidates for infection imaging with PET [7].

*S. aureus* tends to form biofilms, especially on catheters, artificial heart valves, bone, joint prostheses, and bone sequesters [8]. Biofilms reduce the effect of the immune response and antibiotics. As previously described, we thus studied the applicability of two ^68^Ga-labeled phage-display selected dodecapeptides with an affinity towards *S. aureus* biofilm: DOTA-K-A9 and DOTA-GSGK-A11 in our juvenile pig osteomyelitis model [9].

We have previously used ^99*m*^Tc-label methylene diphosphate ([^99*m*^Tc]Tc-DPD) to examine bone remodeling in pigs. We did not observe any tracer uptake in the osteomyelitis lesions [10]. We, therefore, moved on to use [^18^F]NaF in the present study because [^18^F]NaF though acting through similar uptake mechanisms as [^99*m*^Tc]Tc-DPD, [^18^F]NaF is supposed to have a two-fold higher uptake in bones and a better sensitivity for metastatic osteoblastic metastases [11].

Antimicrobial peptides, such as ubiquicidin, can distinguish between mammalian and bacterial or fungal cells and may be targeting vector-candidates for molecular imaging due to their selectivity for bacterial cell membranes in the innate immune system response [12]. We used a [^68^Ga]Ga-ubiquicidin as a bacteria-specific imaging probe.

We also examined the vascular adhesion protein 1 (VAP-1)-targeted PET tracer [^68^Ga]Ga-DOTA-Siglec-9, as an alternative to the traditional tracers for detecting infections. Sialic acid-binding immunoglobulin-like lectin 9 (Siglec-9) is a natural ligand for VAP-1 and is expressed on monocytes and neutrophils and is involved in leukocyte trafficking [13].

## 2. Results

### 2.1. Animal Model

Forty-five OM lesions developed in 15 pigs and none developed in the non-inoculated left control hind limb (Table 1). Bacterial culturing and histopathology confirmed the presence of the inoculated *S. aureus* strain S54F9 in the OMs.

### 2.2. Tracers

#### [^68^Ga]Ga-DOTA-K-A9 and [^68^Ga]GaDOTA-GSGK-A11

**Figure 1:**
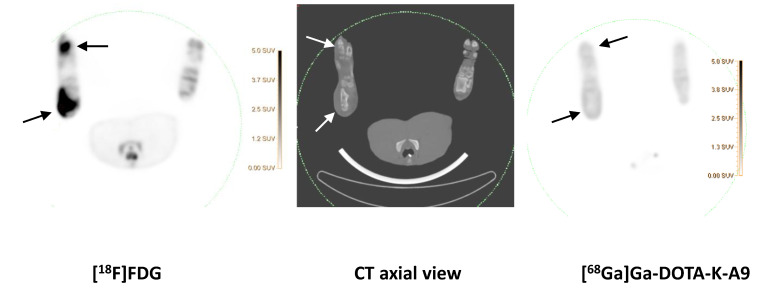
[^68^Ga]Ga-DOTA-K-A9 compared to [^18^F]FDG.

**Figure 2:**
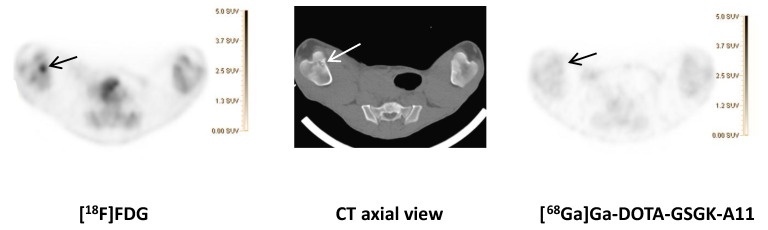
[^68^Ga]Ga-DOTA-GSGK-A11 compared to [^18^F]FDG.

**Figure 3:**
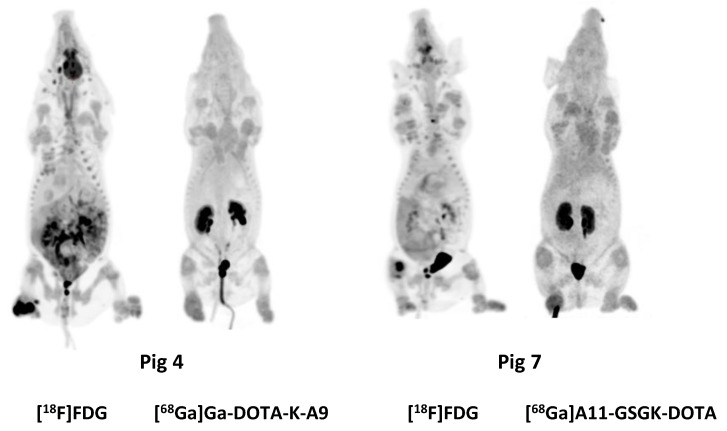
Maximum intensity projections (MIPs) of [^18^F]FDG, [^68^Ga]Ga-DOTA-K-A9, and [^68^Ga]Ga-DOTA-GSGK-A11.

While previous evaluation in murine subcutaneous *S. aureus* infections showed uptake of [^68^Ga]Ga-DOTA-K-A9 [9,14], we saw no increased tracer activity of the two *S. aureus* phage displayed selected peptides, [^68^Ga]Ga-DOTA-K-A9 (Figure 1) and [^68^Ga]Ga-GSG-KA-11 (Figure 2) in 9 and 5, respectively, porcine OM lesions. Figure 3 shows the bio-distribution of the peptides in pigs. Both peptides were excreted by the liver and kidneys.

[^18^F]FDG (left) and Ga-DOTA-K-A9 (right) accumulation is shown in an OM lesion in the right calcaneus and distal II metatarsus of pig 4 (indicated by arrows). The lesions show sequester formation and lysis of the cortical bone on CT in the axial view (middle). Comparable SUV scales are shown to the right of the PET images.

[^18^F]FDG (left) and [^68^Ga]Ga-DOTA-GSGK-A11 (right) accumulation in the right distal femur of pig 7. An OM lesion close to the medial part of the growth zone of the distal right femur with sequester formation and lysis of the cortical bone was revealed on the axial CT image (middle). Comparable SUV scales are shown to the right of the PET images.

MIPs of the [^18^F]FDG, [^68^Ga]Ga-DOTA-K-A9, and [^68^Ga]Ga-DOTA-GSGK-A11.

CT images of two OM in the distal right femur in pig 9 in the axial view. [^68^Ga]Ga-ubiquicidin and [^18^F]NaF compared to [^18^F]FDG accumulation in the same lesions. Comparable SUV scales are shown to the right of the PET images. For a demonstration of the uptake of [^18^F]NaF in the growth zones of long bones, a wider SUV-scale is used in the last picture. The CT image shows sequester formations and lysis of the cortical bones.

### 2.3. [^18^F]NaF

**Figure 4:**
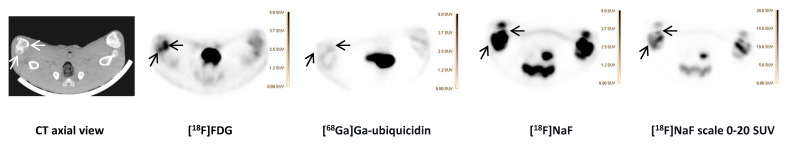
[^68^Ga]Ga-ubiquicidin and [^18^F]NaF compared to [^18^F]FDG.

**Figure 5:**
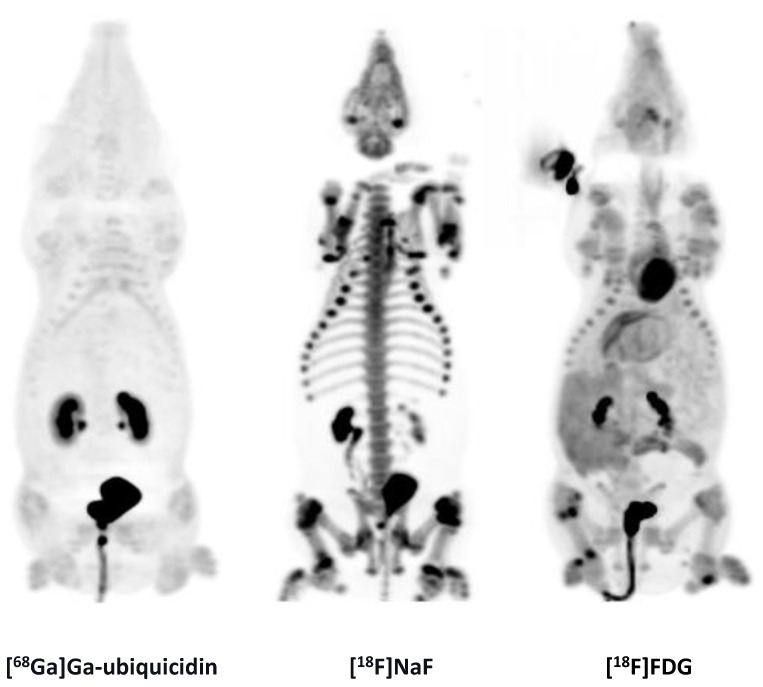
MIPs of [^68^Ga]Ga-ubiquicidin, [^18^F]NaF, and [^18^F]FDG.

We saw no accumulation of [^18^F]NaF in 15 OM lesions (Figure 4). [^18^F]NaF was distributed to the growth zones of the bones (Figure 4) and excreted by the kidneys (Figure 5).

### 2.4. [^68^Ga]Ga-Ubiquicidin

We saw no accumulation of [^68^Ga]Ga-ubiquicidin in 12 OM lesions. [^68^Ga]Ga-ubiquicidin was excreted by the kidneys (Figure 5).

MIPs of the [^68^Ga]Ga-ubiquicidin, [^18^F]NaF, and [^18^F]FDG distribution in pig 9.

### 2.5. [^68^Ga]Ga-DOTA-Siglec-9

**Figure 6:**
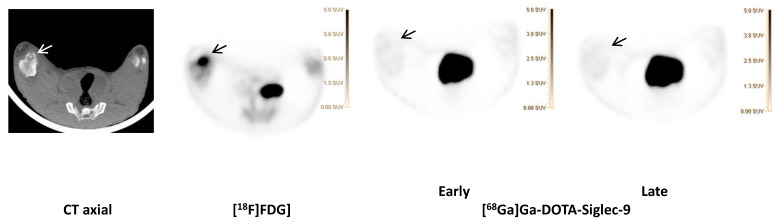
[^68^Ga]Ga-DOTA-Siglec-9 compared to [^18^F]FDG].

**Figure 7:**
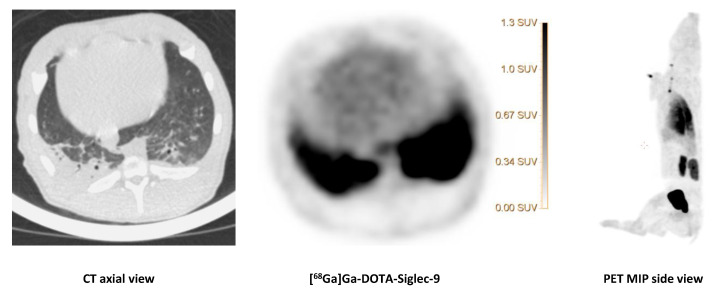
[^68^Ga]Ga-DOTA-Siglec-9 uptake in the lungs.

**Figure 8:**
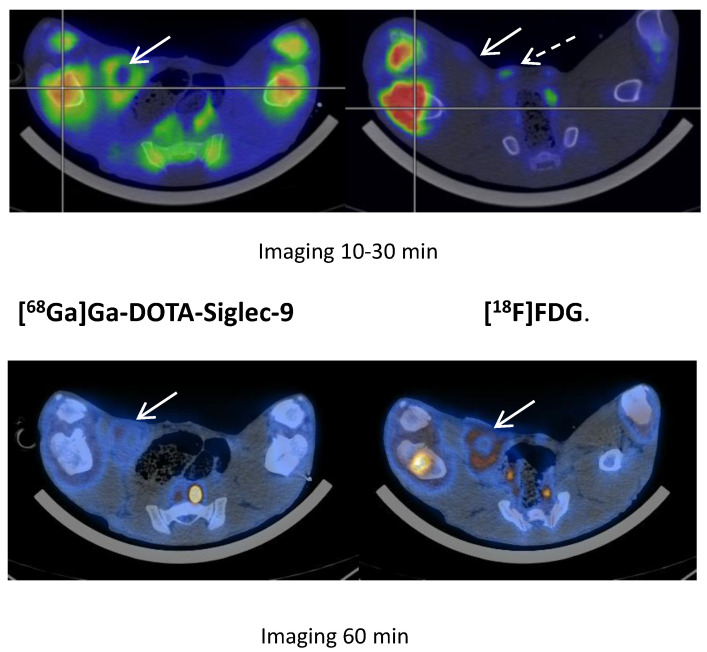
[^68^Ga]Ga-DOTA-Siglec-9 compared to [^18^F]FDG.

We saw no tracer activity neither in the early nor the late images of [^68^Ga]Ga-DOTA-Siglec-9 in 19 osteomyelitis lesions (Figure 6). Instead, the tracer accumulated in dorsocaudal parts of both lungs of, for example, pig 1 (Figure 7). The CT image showed signs of infectious foci in dorsocaudal parts of both lungs. We noticed, however, a general tendency of [^68^Ga]Ga-DOTA-Siglec-9 accumulation in the dorsocaudal parts of the lungs. We also observed this in pigs that did not develop OM or pulmonary infections (data not shown). Figure 8 demonstrates in images acquired 10 to 30 min after tracer injection, a marked ^68^Ga]Ga-DOTA-Siglec-9 uptake in the margin of an inguinal abscess adjacent to the *S. aureus* inoculation site. In the static images acquired after 60 min, the activity of [^68^Ga]Ga-DOTA-Siglec-9 decreased. Whereas the activity of [^18^F]FDG increased slightly in the margin of the abscess (Figure 8).

[^18^F]FDG and [^68^Ga]Ga-DOTA-Siglec-9] (early and late acquisition; 1 and 2 h) uptake in osteomyelitis lesion in the right medial condyle of the right distal femur (indicated by an arrow) of pig 1 and the corresponding CT (axial view). Comparable SUV scales are shown to the right of the PET images.

The CT image (axial view) of the dorsocaudal parts of the lungs of pig 1 showed signs of infection and partial atelectasis (arrow) and the corresponding [^68^Ga]Ga-DOTA-Siglec-9 uptake in the corresponding anatomical area. Axial views of CT and PET and a MIP (side view) of [^68^Ga]Ga-DOTA-Siglec-9 distribution in the pig’s body. The SUV scale bar is shown in the axial PET image.

Pig 12 demonstrated [^68^Ga]Ga-DOTA-Siglec-9 uptake in 5 × 3 × 2 cm chronic inoculation abscess (arrow) in the right inguinal region (10 to 30 min after tracer injection; this is an average image of the frames covering 10 to 30 min, simulating a static image 10 to 30 min) and the uptake of [^18^F]FDG in the same region. Note the similar uptake of [^68^Ga]Ga-DOTA-Siglec-9 in the growth zones of the distal femur and proximal tibia bones and the intense uptake of [^18^F]FDG in the OM lesions of these bones (with sequesters and fistulas formations) in the right hind limb. A hyperplasic medial iliacus lymph node (broken arrow) on the right site had increased [^18^F]FDG uptake compared to the lymph node in the non-infected left hind limb. In the second image, the acquisition was at 60 min post-injection of tracers. The activity in the abscess had decreased for [^68^Ga]Ga-DOTA-Siglec-9 and had increased for [^18^F]FDG.

### 2.6. [^18^F]FDG

[^18^F]FDG accumulated distinguishably in 45 of the 45 OM lesions.

## 3. Discussion

A concern for the health authorities is the increasing MDR in society and its negative effect on morbidity and mortality. Correct antibiotic use in both humans and animals reduces the threat of antibiotic resistance. Timely and specific diagnosis of infectious diseases is essential for the patient’s outcome. Blood cultures can detect invading microorganisms but cannot discriminate between sterile inflammatory and bacterial infections in tissues. Imaging can reveal infection in the body, and early imaging may help to direct the choice of antibiotic treatment and justify continued treatment by antibiotics. Nuclear medicine techniques can visualize the whole body but the available radiopharmaceuticals, such as [^18^F]FDG, are, however, not capable of distinguishing between sterile inflammation and bacterial or fungal infections. Osteomyelitis is a severe bone infection, and the overall purpose of our project was to identify radiotracers that may improve the scanning of bone infection. For diagnosing OM, [^18^F]FDG is usable but unspecific, especially in adults. Therefore, we searched for radioactive tracers for nuclear imaging fitted for differentiation between sterile and septic inflammation to improve the diagnostic accuracy and thus the treatment of, for example, prosthesis-related infections.

We used tracers prepared for targeting the *S. aureus* biofilms or the infected bone lesion itself. We used a well-characterized juvenile porcine model, where OM was selectively induced by an also well-characterized *S. aureus* strain in one hind limb leaving the contralateral hind limb for comparison. Worldwide, *S. aureus* is involved in most cases of hematogenously spread OM. Hematogenously spread OM most often affects children and elderly patients. Our juvenile porcine model has previously demonstrated to mimic osteomyelitis in children [15].

When *S. aureus* enters the skin, neutrophils and macrophages migrate to the site of infection. *S. aureus* avoids this imminent attack by the immune system of the host, for example, by blocking chemotaxis of leukocytes, sequestering host antibodies, hiding from detection via polysaccharide capsule, or biofilm formation, and resisting destruction after ingestion by phagocytes. Bacteriophages are viruses that show no specificity for mammalian cells and infect bacteria exclusively [16]. Most bacteriophages have specificity toward a single bacterial strain. The binding mechanism consists of the attachment of the phages to specific surface receptors or domains located on the surface of the bacterium, subsequently transferring their genetic material into the host cell dedicated for phage replication/reproduction. We have previously examined two ^68^Ga-labeled phage-display selected peptides with affinity for *S. aureus* biofilm and evaluated their potential as bacteria-specific PET imaging agents [9,14]. However, in the present study, we saw no increased accumulation of neither [^68^Ga]Ga-DOTA-K-A9 nor [^68^Ga]Ga-DOTA-GSGK-A11 in the porcine OM lesions, indicating that either no biofilm had formed one week after the inoculation of *S. aureus* or the tracer did not bind to the biofilm in vivo, or was metabolized before the static scan.

The static imaging was for logistic and financial purposes acquired in the same set up as the dynamic scans. We have not analyzed the dynamic scans yet. We, therefore, do not know the kinetics of these two tracers in juvenile pigs. The static scan 60 min after tracer-injection may have been obsolete.

The PET tracer [^68^Ga]Ga-DOTA-Siglec-9 binds to a protein involved in leukocyte extravasations (vascular adhesion protein 1, VAP-1). Only recently, we have analyzed the dynamic data [13]. The uptake of [^68^Ga]Ga-DOTA-Siglec-9 had reversible kinetics and could be modeled with the rev2TCM (4 k-parameters). We demonstrated that [^68^Ga]Ga-DOTA-Siglec-9 was metabolized very quickly and no increased [^68^Ga]Ga-DOTA-Siglec-9 uptake was seen in OM [13]. However, the distribution volume for the uptake of soft tissue infections was elevated, concluding the tracer has a role for soft tissue infections, but not for bone infections (osteomyelitis). In the present study, we also observed an affinity of [^68^Ga]Ga-DOTA-Siglec-9 for infected soft tissue (Figure 7 and Figure 8), although the tracer was present for a short period in this infectious compartment (Figure 8). We can conclude that static scanning should have been earlier when visualizing infections in soft tissues, as the tracer turned out to be reversible [13]. The reason for uptake in the lungs (Figure 7) may be due to an infection, or it may be a result of tracer-trapping in the pulmonary tissue, as the whole-body biodistribution of [^68^Ga]Ga-DOTA-Siglec-9 showed, in general, a significant uptake or accumulation in the lungs, also in non-infected lungs. Jaakkola and coworkers demonstrated that VAP-1 was present in porcine lung endothelium [17]. The tracer accumulation in the lungs may be an effect of high constitutional expression of the receptor, perhaps as a consequence of the lungs being the “excretion organ” for neutrophils. The lungs of pigs have much of the same filter-like function as the spleen in humans. Or it may be an effect of the pulmonary intravascular macrophages in pigs [18].

Theoretically, highly infection-specific radiopharmaceuticals targeting antimicrobial peptides such as the human antimicrobial peptide ubiquicidin seemed to be a worthy approach. The peptide is in mammals, birds, amphibians, and insects, and it protects these animals against infection [19]. We used the small UBI 29-41 synthetic peptide, derived from humans. This UBI binds preferentially to bacteria in vitro and not to activated leukocytes, and can distinguish bacterial infections from inflammation with greater specificity than other UBI peptides [19,20,21,22]. We saw, however, no increase in [^68^Ga]Ga-ubiquicidin accumulation in the *S. aureus* induced OM lesions in our study. The [^68^Ga]Ga-ubiquicidin, though human-derived, should not be species-specific. Also, the *S. aureus* strain we used for inoculation in our animal model was isolated from human pneumonia. Perhaps the mice or rabbit models, inoculated with a human-derived *S. aureus* strain, is not comparable to a pig model. Healthy pigs may eliminate bacterial infections quickly and, thus, bacteria in the pigs may not be detectable with ^68^Ga-labeled UBI 29-41. But it may not exclude the tracer for usage in humans. We have not analyzed the dynamic scans yet. We, therefore, do not know the kinetics of this tracer.

It would have been optimal to analyze the tracer-kinetics before performing the static scans. The kinetic analyses can indicate the optimal times to scan after the tracer-injections. The dynamic scans were performed first and lasted one hour, and consequently delaying the static scans, but the delay may not have been significant as we used [^18^F]- and [^68^Ga]-marked tracers.

*S. aureus* can also invade osteoblasts and can form small-colony variants in the intracellular compartment, where they can survive in a metabolically inactive state while preserving the integrity of the host cell [23,24]. We do not know if this was the case. And if it was, if this was the explanation for the missing sign of osteoblast activity by both [^99*m*^Tc]Tc-DPD and [^18^F]NaF. Another reason could be that the infection was in its early phase before osteoblast recruitment and reparative processes took place. [^18^F]NaF diffuses into the extravascular fluids of bones and is incorporated in hydroxyapatite. Thus, the uptake of [^18^F]NaF reflects both blood flow and bone remodeling. We have recently demonstrated only slightly increased blood perfusion in the infected limb compared to the not infected limb [25]. This amount of blood perfusion may not have been sufficient to increase the [^18^F]NaF accumulation. It was, however, not ethically justifiable for us to keep the pigs alive for more than seven days after they had established the *S. aureus* infection as human endpoints were reached. This model, therefore, reflected acute/subacute OM.

[^18^F]FDG accumulated very distinguishably in the OM in our juvenile pig model, and even small lesions were recognizable on CT images alone. The same may be the case in children as juveniles have fewer concurring diseases and seldom degenerative changes causing inflammation of the bone and joint tissues. [^18^F]FDG secures that no OM is unnoticed. We have previously addressed a possible reduction of [^18^F]FDG activity in children [26].

The overall osteomyelitis project used an ambitious protocol as we scanned each pig several times, using a combination of scanning techniques (SPECT/CT and PET/CT) and series of tracers from 18 to 20 hours. We scheduled the scan protocol so that a new scan was not initiated until at least five half-lives after the latest PET-tracer had passed. The exceptions occurred in some of the pigs when the ^68^Ga-labeled tracer followed by the ^18^F-labeled tracers. It was impractical to wait the full 5 × 67.7 min from the injection of ^68^Ga-labeled tracer to [^18^F]FDG scan. Instead, close to four half-lives passed, and the injected activity of [^18^F]FDG was at least double the injected activity of the ^68^Ga-based tracers. The pigs were euthanized after the last scan and transported for necropsy at noon the following day; therefore, it was possible to raise the injected activity for the final scan. Due to the expected shorter half-lives and slightly slow uptake in OM, we gave more [^68^Ga]Ga-Ubiquicidin. We would have liked to administrate the same amount as of [^68^Ga]Ga-DOTA-Siglec-9 but due to the fractionate and usage of only half the activity, we were only able to synthesize half as much [^68^Ga]Ga-Ubiquicidin as [^68^Ga]Ga-DOTA-Siglec-9. Regarding the radio-labeled phage displayed selected peptides, [^68^Ga]Ga-DOTA-K-A9 and [^68^Ga]Ga-DOTA-GSGK-A11, we injected the maximum amount of achieved radiotracer. The main reasons for varying injected activities were decreasing yield from ^68^Ga-generator, changing ^68^Ga-labeling yield (especially for A11 peptide [9]), and different delays between the time of production to the time of injection. In general, it is not easy to guess the adequate and optimal injectable radioactivity, so we tried variable activities.

## 4. Materials and Methods

### 4.1. Animal Model

Fifteen domestic pigs, all clinically healthy, specific pathogen-free Danish Landrace Yorkshire cross-bred female pigs aged 8–9 weeks weighing 18.3–24.5 kg, were purchased from local commercial pig farmers. After at least one week of acclimatization, the pigs fasted over-night. Then they were premedicated with s-ketamine (Pfizer, Ballerup, Denmark) and midazolam (B. Braun Medical, Frederiksberg, Denmark) intramuscularly and anesthetized with propofol (B. Braun Medical, Frederiksberg, Denmark) intravenously. Finally, they were inoculated with a well-characterized porcine strain of *S. aureus* S54F9 (approximately 10,000 CFU/kg body weight) into the femoral artery of the right hind limb to induce OM in that limb, as described elsewhere [18]. After the onset of clinical signs, for example, limping of the right hind limb, redness, and local swelling of the leg, starting typically on day 3–4 after inoculation, the pigs were supplied with intramuscular procaine benzylpenicillin (10,000 IE)/kg procaine benzylpenicillin (Penovet, Boehringer Ingelheim, Copenhagen, Denmark) once daily until 48 h before scanning [27]. The pigs were all treated with 30–45 µg/kg buprenorphine (Indivior, Berkshire, United Kingdom) intramuscularly thrice daily from the time before inoculation and until scanning at day 6 and 7 after inoculation [27]. After scanning, pigs were euthanized with an overdose of pentobarbitone (Scan Vet Animal Health, Fredensborg, Denmark) while still under anesthesia. Humane endpoints were: anorexia for more than 24 h, superficial respiration, the inability to stand up, or signs of systemic infection. After the inoculation, pigs were housed in separate boxes with soft bedding, fed twice daily with a restricted standard pellet diet (DIA plus FI, DLG, Denmark), and had ad libitum access to tap water. The environment was characterized by a room temperature of 20 °C, relative humidity of 51–55%, 12 h in light/12 h in dark cycles, an exchange of air at least eight times per hour. Pigs were fasted overnight before anesthesia.

Osteomyelitis was verified by CT imaging, necropsy, histology, and/or bacterial cultivation.

The study was approved by the Danish Animal Experimentation Board, license no. 2012-15-2934-000123 and 2017-15-0201-01239 in accordance with 2010/63/EU. All facilities were approved by the Danish Occupational Health Surveillance.

### 4.2. Tracers

#### 4.2.1. *S. aureus* Biofilm (Phage Selected Peptides ^68^Ga-labeled DOTA-K-A9 and DOTA-GSGK-A11)

The selection, radio-synthesis, the in vitro binding, and preliminary in vivo evaluation of the two phage display selected DOTA-peptide derivatives with an affinity towards *S. aureus* has previously been described [9,14]. Briefly, in the ^68^Ga-labeling of DOTA-K-A9, as described in [14], the ^68^GaCl_3_ from the generator was trapped on a Varian SCX cartridge and eluted with a 5 M NaCl/5.5 M HCl (2.5%) solution (700 µl). The following ^68^Ga-labeling was achieved by mixing the pre-purified ^68^GaCl_3_ with the precursor DOTA-K-A9 (70 µg, 37 nmol) in 0.1 M HEPES solution (5 mL) pH 6.5 adjusted with 30% ultrapure HCl and heated at 95 °C for 3.5 min. The post-purification of [^68^Ga]Ga-DOTA-K-A9 was performed using a C18-light cartridge, preconditioned with 50% EtOH in water solution (5 mL) and 0.1 M HEPES (5 mL) pH 5.2 adjusted with 30% ultrapure HCl. The [^68^Ga]Ga-DOTA-K-A9 reaction mixture was immediately transferred and trapped on a C18-light cartridge and rinsed with the 0.1 M HEPES pH 5.2 (2 mL) before it was eluted with 50% EtOH in water solution (1 mL). The final formulation (9 mL) of the product was achieved by adding isotonic saline (8 mL). The product formulation was further diluted prior to in vivo testing, to achieve an EtOH content <5% (*v*/*v*). Synthesis time: around 30 min. RCY: 56 ± 4%. RCP: 93 ± 1% EOS and 90 ± 1% at 2.5 h. pH: 5.1–5.7.

The ^68^Ga-labeling of DOTA-GSGK-A11, as described in [9], was achieved by mixing the precursor DOTA-GSGK-A11 (70 µg, 34 nmol) in 0.1 M HEPES (5 mL) pH 6.5 adjusted with 30% ultrapure HCl and adding the pre-purified ^68^Ga-eluate trapped on a Varian SCX cartridge and eluted with a 5 M NaCl/5.5 M HCl (2.5%) solution (700 µl). The mixture was heated at 95 °C for 7 min before purification was performed using a C18-light cartridge preconditioned with a 50% EtOH in water/sodium phosphate (2%) solution (5 mL) and isotonic saline (5 mL). After end heating, [^68^Ga]Ga-DOTA-GSGK-A11 was immediately trapped on the C18-light cartridge, while non-chelated ^68^Ga passed through. The cartridge was rinsed with isotonic saline (2 mL) before the purified [^68^Ga]Ga-DOTA-GSGK-A11 was eluted by employing a 50% EtOH in water/sodium phosphate (2%) solution (1 mL). The final formulation of the product for biological testing was achieved by adding isotonic saline (5 mL). Synthesis time: around 30 min. RCY: 69 ± 2%. RCP: 96 ± 3% EOS and 96 ± 2% at 2 h. pH: 6.5–7.0.

The average injected activity was a median of 760 MBq (range 348–848 MBq) of [^68^Ga]Ga-DOTA-K-A9, corresponding to ~17–46 MBq/kg and median 475 MBq (range 65–500 MBq) of [^68^Ga]Ga-DOTA-GSGK-A11, corresponding to ~3–23 MBq/kg. PET was performed median ~60 min (60–61min) after injection of [^68^Ga]Ga-DOTA-K-A9 (pigs 4–6, Table 1) and ~62 min (62–62 min) after injection of [^68^Ga]Ga-DOTA-GSGK-A11 (pigs 7–8, Table 1) in the jugular vein.

#### 4.2.2. Bone Remodeling ([^18^F]NaF)

Fluoride-18 was produced via the ^18^O (p,n) ^18^F nuclear reaction using a cyclotron. The target content was passed through a QMA-light Sep-Pak (Waters, Hedehusene, Denmark) to trap F-18. The solution was washed with 10 mL sterile water for injection (10 mL) to remove any residual [^18^O]H_2_O and then dried with an argon stream. [^18^F]NaF was set free by eluting the QMA Sep-Pak with 10 mL NaHCO_3_ and 10–20 mL air into the product vial. The average injected activity was as median 184 MBq (range 165–196 MBq), corresponding to ~8–10 MBq/kg. The PET was performed ~57 min (33–78 min) after injection of [^18^F]NaF (pigs 9–13, Table 1).

#### 4.2.3. Bacterial Cell Membranes ([^68^Ga]Ga-Ubiquicidin)

NOTA-Ubiquicidine 29-41 acetate was obtained from ABX, Radeberg, Germany. Ultrapure Water (metal-free water) and ultra-pure hydrochloric acid (30%) was obtained from Merck (Merck KGaA, Darmstadt Germany) and cation exchange cartridge Strata XC 10, Phenomenex Inc., Værløse, Denmark (Strong cation exchange) was used. Sterile water and sterile isotonic saline solution (9 mg/L) were purchased from the Hospital Pharmacy in Aalborg. Sodium acetate was of lab-grade from Sigma Aldrich (St. Louis, MI, USA). The synthesis was inspired by the synthesis published by Ebenhan et al. [20] and Vilche et al. [28]. Briefly, Ga-68 was obtained by eluting a ^68^Ge/^68^Ga generator (IGG100, Eckert & Ziegler AG Eurotope GmbH, Berlin, Germany). The generator eluate was fractionated and the 0.7 mL fraction with the highest radioactivity was collected and used for the synthesis. In our case, this was the fraction from 1. 6–2.3 mL (7 −/+ 2% of the radioactivity came in the early fraction 0–1.6 mL, 50 −/+ 2% came in the fraction we used, and 41 −/+ 2% came after (2.3–10 mL)). A total of 625 µL of the 0.7 mL eluate was mixed with 30 µL sodium buffer solution (5 M, pH 5.5) and 600 µL of this mixture was transferred into a reactor containing NOTA-Ubiquicidin 29-41 acetate (400 µg, 202 nmol, dissolved in 64 µL metal-free water) resulting in an overall concentration of NOTA-Ubiquicidin 29-41 of 0.3045 mM. The reaction vial was placed in a water bath at a temperature of 90 °C for 12 min. Sterile water (6 mL) was added. The mixture was run through a cation exchange cartridge Strata XC 10. The reaction vial was washed with water (2 mL), and the wash was also run through the cation exchange cartridge. Approximately 80% of the reaction mixture was trapped on the cation exchange cartridge; approximately 17% stayed in the reactor, and 3% went through the cation exchange cartridge. The product was released from the cation exchange cartridge by 0.5 mL 60% EtOH in sterile water, followed by 5 mL sterile saline, leaving 8% of the radioactivity on the cation exchange cartridge. The synthesis took 18–20 min, and the labeling yield was 162–203 MBq depended on how strong the Ge/Ga generator was (72–74% d.c). The specific radioactivity was ~1 MBq/nmol, assuming that all the NOTA-Ubiquicidin 29-41 applied in the synthesis ends up in the product vial. The radiochemical purity of [^68^Ga]Ga-Ubiquicidin was examined using the setup described by Vilche et al. [17]. Briefly, the radio-HPLC system consisted of Dionex Ultimate 3000 HPLC system (Dionex Denmark) and a NaI radio detector from LabLogic (LabLogic, UK) and C-18 columns (4.6 × 150 mm, 5 µm). The HPLC conditions were as follows: flow rate = 1 mL/min; Channel A = 0.1% TFA/water; Channel B = 0.1% TFA/acetonitrile. Gradient: during 0−6 min, from 100% A to 40% A; Cfrom 6–10 min isocratic 40% A. The radiochemical purity was higher than 96% in all our syntheses.

The average injected activity was median 117 MBq (range 109–120 MBq), corresponding to ~5–6 MBq/kg. The PET was performed ~67 min (66–69 min) after injection of [^68^Ga]Ga-Ubiquicidin (pigs 9–11, Table 1).

#### 4.2.4. Leukocyte Trafficking ([^68^Ga]Ga-DOTA-Siglec-9)

The radioactive labeling of DOTA-Siglec-9 was previously discussed and described in detail [13,29]. Briefly, ^68^Ga was eluted from a *GalliaPharm*^®^^68^Ge/^68^Ga generator (Eckert & Ziegler, Radiopharma GmbH, Berlin, Germany) using aq HCl (10 mL, 0.1 M). Gallium was trapped on a cation exchange cartridge (Strata-X-C 33 u Polymeric Strong, Phenomenex, Værløse Denmark), and released from the cartridge with an acidified acetone solution (0.6 mL, made from ultrapure HCl (30%, 0.21 mL) and sterile water (2.23 mL) in a total volume of 100 mL with acetone) from the SCX cartridge into a preheated reaction vial (100 °C). The acetone contents were reduced by azeotrope evaporation (100 °C, the addition of HCl (0.5 mL, 0.1 M) after 1.5 min, metal-free water (0.5 mL) after 2.5 min and HEPES buffer (124.0 ± 2.0 mg in 500 μL metal-free water) after 5.5 min). Followed by an addition of the DOTA-Siglec-9 peptide (40.0 μg, 16.5 nmol, dissolved in 200 μL of metal-free water. The reaction mixture was heated for 5 min for the ^68^Ga incorporation to take place before it was quenched by adding sterile water (10 mL). The product mixture was then run through a preconditioned C-18 Sep-Pak cartridge (Waters, Hedehusene, Denmark) to trap [^68^Ga]Ga-DOTA-Siglec-9 (preconditioned with 67% EtOH/33% sterile water (10 mL) followed by sterile saline (10 mL)).

The product ready for injection, [^68^Ga]Ga-DOTA-Siglec-9, was released from the cartridge with ethanol (67%, 0.5 mL) followed by saline (9 mL) directly into the product vial through a sterile filter.

The radiochemical purity of [^68^Ga]Ga-DOTA-Siglec-9 was determined using a radio detector coupled with a reversed-phase high-performance liquid chromatography (radio-HPLC) (Jupiter C18 column, 4.6 × 150 mm, 300 Å, 5 μm; Phenomenex, Torrance, CA, USA). The HPLC conditions were as follows: flow rate = 1 mL/minute; λ = 274 nm; Channel A = 0.1% TFA/water; Channel B = 0.1% TFA/acetonitrile; gradient: during 0−2 min, 82% A and 18% B; during 2−11 min, from 82% A and 18% B to 40% A and 60% B; during 11−15 min, from 40% A and 60% B to 82% A and 18% B; during 15−20 min, 82% A and 18% B. The radio-HPLC system consisted of Dionex Ultimate 3000 HPLC system equipped with a 4-wavelength simultaneous data collector (Dionex Denmark) and of a NaI radiodetector from LabLogic (LabLogic, UK). The overall yield of the reaction was approximately 62% ndc with a reaction time of approximately 25 min. The molar activity was 35 ± 10 MBq/nmol.

The average injected activity was as median 178 MBq (range 126–253 MBq), corresponding to ~8–12 MBq/kg. The PET was performed ~72 min (60–126 min, early scan) and 139 min (118–186 min, late scan) after injection of [^68^Ga]Ga-DOTA-Siglec-9 (pigs 1–3 and 12–15, Table 1).

#### 4.2.5. Glucose Metabolism ([^18^F]FDG)

^18^F were produced at the PET Center Aarhus using either a PETtrace 800 series cyclotron (GE Healthcare, Uppsala, Sweden) or a Cyclone 18/18 cyclotron (IBA, Louvain La Neuve, Belgium). [^18^F]FDG was produced by a standard procedure applying a GE Healthcare MX Tracerlab synthesizer, Mx cassettes supplied by Rotem Industries (Arava, Israel), and chemical kits supplied by ABX GmbH (Radeberg, Germany). Radiochemical purity was higher than 99%. The median injected activity was 125 MBq (range 94–497 MBq), corresponding to ~5–24 MBq/kg. The PET was performed ~61 min (50–76 min) after injection of [^18^F]FDG (pigs 1–15, Table 1).

### 4.3. CT and PET

The pigs were scanned at locations; Aalborg and Aarhus PET Centers. The pigs were carefully placed in dorsal recumbency to make sure the infected and not infected limb would be comparable in CT images. They were in propofol anesthesia and mechanically ventilated throughout the scan. Reflexes, pulse, oxygen saturation, and body temperature were monitored. After unenhanced CT for attenuation correction and anatomical co-registration, whole-body static PET imaging was performed with 5 min per bed position in three-dimensional mode. Pigs are not designed for erect walking, and their limbs cannot be stretched in the same manner as human extremities. Despite that, we kept the standards for imaging of humans; views in axial, coronal, and sagittal planes. But in the thorax, we changed the directions to cranial and caudal.

In Aalborg, to evaluate if any lytic lesions had developed, the pigs had a diagnostic CT scan (GE VCT Discovery True 64 PET/CT scanner 2006, GE Healthcare, Chicago, Illinois, USA).

The scan fields covered 15 cm in the axial direction. PET images were reconstructed using an ordered subset expectation maximization (OSEM) iterative algorithm (3D ViewPoint algorithm, GE Healthcare). The reconstruction parameters were 2 iterations, 28 subsets, 128 × 128 matrix in 47 slices, (5.5 × 5.5 × 3.3) mm^3^ voxel size, and a 6 mm Gaussian filter.

Due to scanner replacement, later pigs were scanned on a different scanner than the previous pigs. They were scanned on a Siemens Biograph mCT (Siemens, Erlangen, Germany) with time-of-flight (TOF) detection. The scan field covered 22 cm in the axial direction. The images were reconstructed with an OSEM algorithm without using the resolution recovery option (setting “Iterative + TOF”). The reconstruction parameters were 3 iterations, 21 subsets, 400 × 400 matrix in (1.02 × 1.02 × 2.03) mm^3^ voxels, and a 3 mm Gaussian filter. On both scanners, image reconstruction included attenuation-correction based on the CT scan.

In Aarhus, all examinations were performed with Siemens Biograph TruePoint™ 64 PET/CT, Siemens Healthineers, Erlangen, Germany. The pigs were placed in recumbency. Initially, a scout view was obtained to secure body coverage from snout to tail. We used visually comparable PSF reconstruction protocols (TrueX) (four iterations, 21 subsets, 3-mm Gaussian post-processing filter, matrix size 336  ×  336, voxel size (2 mm  ×  2 mm  ×  2 mm).

In the present study, only the whole-body static PET/CT scans are presented.

## 5. Conclusions

We report these negative results as we find them valuable to other research groups developing new tracers for osteomyelitis. They may save other research groups from doing the same experiments and as a platform for the development of new potential tracers, or to use other scanning protocols. We also have an ethical responsibility to report negative results to prevent unnecessary animal studies from being repeated by other researchers.

[^18^F]FDG was the best tracer for the detection of OM in peripheral bones in our juvenile pig model seven days after *S. aureus* induced OM.

## Figures and Tables

**Table 1:** Standard uptake values (SUV) of different radiotracers in OM lesions.

Pig Tracer	1	2	3	4	5	6	7	8	9	10	11	12	13	14	15
[^68^Ga]Ga-DOTA-Siglec-9 Leukocyte trafficking	0.4–0.4	0.4–0.5	0.2–0.5									1.3	0.9–1	0.7–0.9	1
[^68^Ga]Ga-DOTA-K-A9 *S. aureus* (biofilm)				0.7–0.7	0.9–1.2	0.5–0.7									
[^68^Ga]Ga-DOTA-GSGK-A11 *S. aureus* (biofilm)							0.7–0.8	0.2–0.3							
[^68^Ga]Ga-ubiquicidin Bacterial cell membranes									0.6–0.8	0.6–0.9	0.5–0.6				
[^18^F]FDG Glucose metabolism	4–7.3	2.3–5.9	2.9–4.5	10.5–16.3	3.4–9	4–9.2	3–6.4	2.4–8	5.4–5.9	4.4–5.1	1–3.9	6.1	7.8–10.1	4–7.8	3.6
[^18^F]NaF Bone remodeling									9.2–13.6	15.2–17	5.7–19.2	19.8	7.8–17.6		
Number of lesions	3	4	5	2	3	4	3	2	4	3	5	1	2	3	1
